# The Application of Metal–Organic Frameworks in Water Treatment and Their Large-Scale Preparation: A Review

**DOI:** 10.3390/ma17091972

**Published:** 2024-04-24

**Authors:** Yuhang Xie, Teng Zhang, Bo Wang, Wenju Wang

**Affiliations:** 1 Frontiers Science Center for High Energy Material, Beijing Key Laboratory of Photoelectronic Ministry of Education, Advanced Research Institute of Multidisciplinary Science, School of Chemistry and Chemical Engineering, Beijing Institute of Technology, Beijing 100081, China; xyh1654228985@163.com (Y.X.); bowang@bit.edu.cn (B.W.); 2 Conversion Materials, Key Laboratory of Cluster Science, Ministry of Education, Advanced Research Institute of Multidisciplinary Science, School of Chemistry and Chemical Engineering, Beijing Institute of Technology, Beijing 100081, China; 3 Advanced Technology Research Institute (Jinan), Beijing Institute of Technology, Jinan 250300, China; 4 School of Energy and Power Engineering, Nanjing University of Science and Technology, Nanjing 210094, China

**Keywords:** MOFs, wastewater treatment, large-scale preparation, spray drying, high pressure homogenization

## Abstract

Over the last few decades, there has been a growing discourse surrounding environmental and health issues stemming from drinking water and the discharge of effluents into the environment. The rapid advancement of various sewage treatment methodologies has prompted a thorough exploration of promising materials to capitalize on their benefits. Metal–organic frameworks (MOFs), as porous materials, have garnered considerable attention from researchers in recent years. These materials boast exceptional properties: unparalleled porosity, expansive specific surface areas, unique electronic characteristics including semi-conductivity, and a versatile affinity for organic molecules. These attributes have fueled a spike in research activity. This paper reviews the current MOF-based wastewater removal technologies, including separation, catalysis, and related pollutant monitoring methods, and briefly introduces the basic mechanism of some methods. The scale production problems faced by MOF in water treatment applications are evaluated, and two pioneering methods for MOF mass production are highlighted. In closing, we propose targeted recommendations and future perspectives to navigate the challenges of MOF implementation in water purification, enhancing the efficiency of material synthesis for environmental stewardship.

## 1. Introduction

In recent years, pollution has surged, becoming a formidable adversary to freshwater ecosystems, the atmosphere, and human well-being [1,2,3]. The infiltration of heavy metals such as arsenic (III), lead (II), and mercury (II), alongside a variety of organic pollutants ranging from dyes and pesticides to endocrine disruptors and pharmaceuticals, has raised global alarms [4,5,6,7]. It has been demonstrated that that traditional wastewater treatment has many shortcomings. For example, the removal capacity of high-concentration organic pollutants, micro-pollutants, high-salt wastewater and nitrogen and phosphorus is limited, and the resource utilization capacity is weak. Even trace levels of these contaminants remaining in water can cause adverse health effects, including skin damage, infectious diseases, acute or chronic poisoning, and so on [8]. The advent of metal–organic frameworks (MOFs) has opened new horizons in combating these pollutants [9,10,11]. These structures, with their metal nodes interconnected by organic ligands, create a range of porous geometries from one to three dimensions [12,13,14]. Their robust crystalline frameworks offer unmatched porosity and vast surface areas, which have ushered them into a plethora of applications such as gas storage, chemical catalysis, and environmental clean-up, to name a few. The ultrahigh porosity and larger specific surface area of MOFs have significantly contributed to their wide applications in luminescence, gas storage, chemical catalysis, gas adsorption, biomedical imaging, catalytic degradation, energy generation, and environmental remediation [9,14,15,16]. Regarding pollutant removal, recent review pa-pers have detailed the environmental applications of MOFs, primarily focusing on the adsorption of diverse contaminants (e.g., organics, heavy metals, and toxic gases) and catalytic eliminations of various water pollutants through methods such as Fenton-like oxidation [17], photocatalysis [18], electrocatalysis [19], and sulfate radicals-based reactions [20]. These studies consistently propose that MOFs can serve as effective and re-cyclable materials for pollution remediation.

While metal–organic frameworks (MOFs) offer significant benefits, their application in environmental management is not without challenges [21,22,23]. Firstly, the water stability and biocompatibility of MOFs themselves hinder the use of some MOFs on an industrial scale. Secondly, MOFs in powdered form are susceptible to quick depletion when used practically, leading to reduced longevity and higher operational costs. Thirdly creating multifunctional MOFs with precise control over their size, shape, and selectivity is a complex process. Achieving cost-effective, large-scale production for industrial use remains a demanding task requiring further innovation. To leverage the full potential of MOFs and overcome these practical hurdles, innovative large-scale production methods have been introduced. These include electrochemical [24], microwave [25], and mechanochemical [26] techniques, along with newer methods such as spray drying [27], flow chemistry [28], and high-pressure homogenization [29]. These advancements hold promise for refining MOF production and making their exceptional properties more accessible for widespread industrial application. However, currently, the materials suitable for achieving large-scale preparation are still limited, and in the actual application process, the integrity of the large-scale MOF preparation process needs improvement.

**Figure 1:**
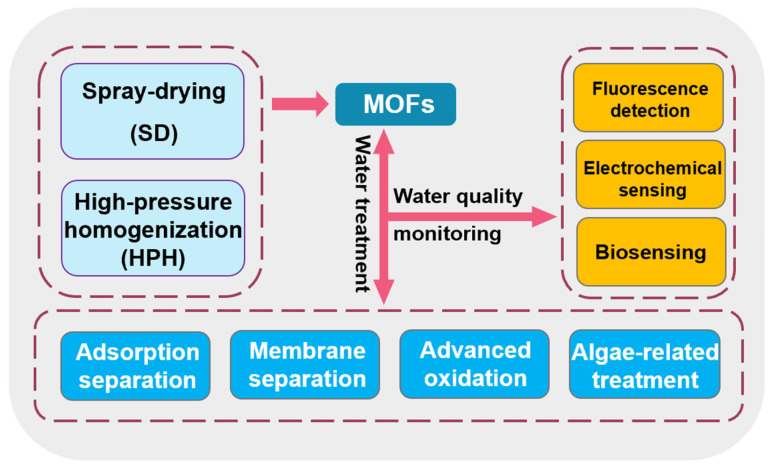
Summary content structure diagram.

Based on this, this review tries to integrate all current MOF-related water treatment methods so that readers can have a comprehensive understanding of this (Figure 1). It is worth mentioning that this also includes the detection technology of MOF-related pollutants, and gives practical recommendations from the raw material production side. In this way, the comprehensive application of MOF in the field of water treatment from production to water quality testing to sewage treatment is promoted.

## 2. MOF-Based Water Treatment Strategy

### 2.1. Separation Method for the Treatment of Pollutants in Water

#### 2.1.1. Adsorption Separation

**Figure 2:**
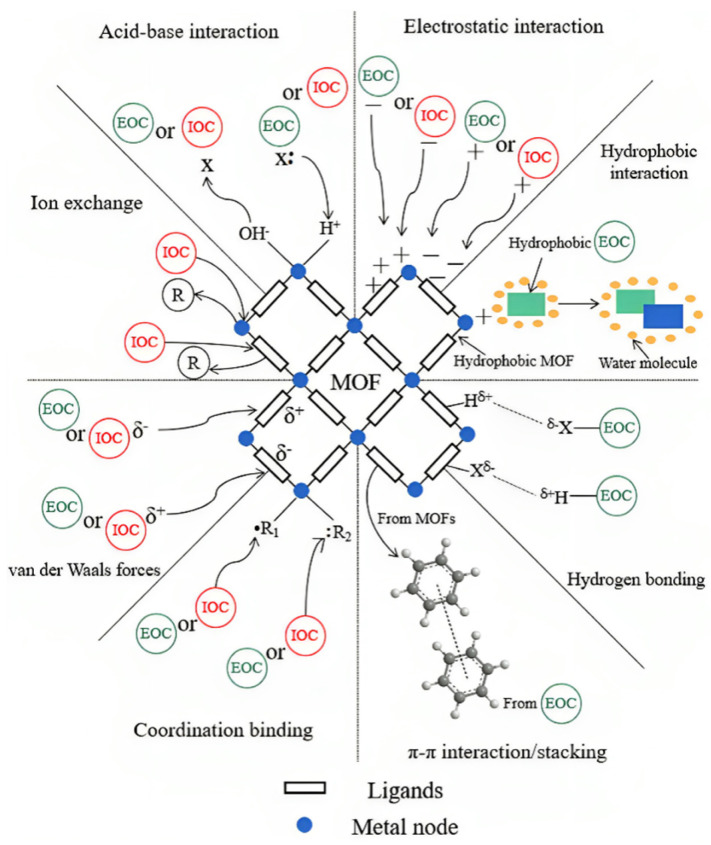
Schematic diagram of possible mechanisms for adsorptive removal of contaminants over MOFs [45].

Adsorption emerges as a leading technique in water pollution control due to its simplicity, cost-effectiveness, and high efficiency [30,31,32]. Nevertheless, traditional adsorbents for inorganic contaminants (IOCs) often suffer from inherent limitations such as inadequate adsorption capacity, non-selective adsorption, and subpar recyclability [33]. To overcome these barriers, innovative adsorbents are being actively pursued. MOFs, with their impressive capabilities, are at the forefront of this research. Four common MOF adsorptive modes for IOCs include van der Waals force, electrostatic interaction, ion exchange, and coordination binding (Figure 2). A notable study by Lv et al. [34] highlighted a hydrothermally synthesized Ni-MOF with a high affinity for arsenic (V), where adsorption capacity was optimally enhanced at 454.94 mg/g by balancing van der Waals forces with the density of oxygen-containing functional groups after pyrolysis at 400 °C. Similarly, the removal of emerging organic contaminants by MOFs involves diverse interactions such as electrostatic, hydrophobic, acid-base, p-p interactions, hydrogen bonding, and coordination binding [35,36,37,38,39,40]. In a remarkable study, Seo et al. [41] utilized the p-p and electrostatic interactions within UiO-66 to capture methylchlorophenoxy propionic acid from water, boasting an adsorption efficiency nearly 30 times greater than that of activated carbon.

And to sum up the latest research, UiO series and MIL series are still the most commonly used MOF systems for adsorption and removal of water pollutants. For example, experimental data in the recent work of D et al. [42] show that the metal–organic skeleton of UiO-66-NH_2_ can effectively remove fluoride ions present in industrial wastewater (using 100 mg of MOF in 50 mL industrial wastewater to remove 70–80% of fluoride in 60 min). The MIL series has also been recently summarized in the related literature [43]. It is important to acknowledge that the widespread adoption of these MOFs in water purification is largely due to their superior water stability. Generally, MOFs exhibit robust water stability under conditions such as the presence of metal ions with large atomic radii and low ionization potentials, or ligands with fewer six-membered rings and additional cyclic bivalent nodes. Furthermore, the advent of machine learning in research offers a new avenue for predicting the water stability of MOFs with greater ease [44].

While MOFs hold promise as superior adsorbents, their transition from laboratory to market is impeded by economic constraints. The raw materials for MOFs are relatively inexpensive, yet commercial procurement costs far exceed those of conventional adsorbents such as activated carbon and zeolite [46,47,48,49,50]. This discrepancy stems from the predominant synthesis technique for MOFs—solvothermal methods—which are notably laborious, reliant on organic solvents, and necessitate intricate purification processes. These factors cumulatively inflate production costs, rendering MOFs less commercially viable. Furthermore, balancing the adsorption efficacy against the manufacturing expense poses an ongoing challenge. Notwithstanding these production hurdles, the sorbent market is witnessing robust growth, as underscored by the “Global Economy of Sorbents 2024” report. This report documents a market expansion from USD 4.67 billion in 2023 to an estimated USD 5 billion in 2024, reflecting a healthy compound annual growth rate of 7.0%. Such growth indicates vigorous efforts to innovate and expand the portfolio of available adsorbents, hinting at the potential for MOFs to capture a share of this expanding market once economical production barriers are surmounted.

#### 2.1.2. Membrane Separation

In addition to adsorption separation, membrane technology, leveraging the principles of size exclusion and adsorption, has emerged as a highly effective and convenient approach for the one-step removal of aquatic contaminants [51]. The integration of MOFs into composite membranes capitalizes on their distinct characteristics—such as tunable modularity, functionality, vast surface area, and porous structure [23]. Various strategies for preparing nanoMOF-based composite membranes are summarized in Table 1. For instance, biocompatible and renewable cellulose is an excellent substrate/matrix for compositing MOFs. Abdelhameed et al. [52] demonstrated this by coating Cu-BTC (1,3,5-Benzenetricarboxylic acid) onto cotton fabrics and applied it for organophosphate insecticide pollution remediation. The Cu-BTC@cotton composite showed a maximum ethion adsorption capacity of 182.0 mg g^−1^ and followed the Langmuir model. In a similar vein, Schelling et al.’s functionalization of cotton fabrics with UiO-66 showcased its efficacy in herbicide adsorption [53]. Table 2 presents a curated selection of 15 research studies from the last one to two years on MOF composite membranes used in water treatment, chosen for their distinctive surface properties. These are intended to provide readers with fresh perspectives and spark innovative developments in the field.

Moreover, most dyes are eliminated onto membranes containing MOFs through electrostatic interactions [54,55,56]. In addition to the advantages of using MOF-containing membranes to eliminate dyes, practical applications still pose a huge challenge due to their stability in wastewater and lack of robustness in complex pollutants present in water environments. In addition, research on the effects of coexisting ions and operating modes on the elimination of dyes in wastewater is very limited. Therefore, in-depth research on the factors and reaction mechanisms that affect dye removal is crucial for enhancing the sustained application of MOF-containing membranes in dye decontamination. Similarly, in terms of removing heavy metal pollutants, membranes containing MOFs have the characteristics of fast equilibrium time, strong removal ability, and high selectivity towards target heavy metal ions, and can maintain excellent performance even under extreme environmental conditions [57,58,59,60]. To our knowledge, the reaction mechanism is mainly attributed to electrostatic interactions and pore-filling effects. However, most current research has focused on selectively eliminating a target ion, and the actual water environment is complex and variable. Therefore, further research on the simultaneous treatment of multiple pollutants by MOF-containing membranes is of great significance for actual wastewater purification.

**Table 1:** Various strategies for the preparation of nanoMOF-based composite membranes.

Membrane Type	Strategy	Unique Characteristics	References
TFN	Integrating MOF nanoparticles within the polyamide (PA) layer is deftly achieved using the interfacial polymerization (IP) process	PA layer can be tailored, affecting factors such as the degree of cross-linking and the layer’s thickness. These modifications have the potential to enhance the layer’s permeability to water and, in certain instances, can also improve its selectivity in terms of solute rejection.	[61]
	Applying a layer of nanoMOFs onto the PA layer	Modifying a membrane’s surface can alter its hydrophilicity and charge, which can introduce or enhance antifouling and antibacterial properties. Such enhancements are integral to developing durable membranes that maintain high separation efficiency over extended periods.	[62]
MMM	Embedding MOF nanoparticles into the matrix of a substrate or a single-layer membrane	Adjusting the fabrication parameters of a membrane offers the potential to enhance its porosity and reduce both its thickness and the complexity of its internal pathway, known as tortuosity. These modifications could lead to a reduced structural parameter (S-value) and an increased water flux, thereby optimizing the membrane’s filtration performance.	[63]
Others	Incorporating water unstable MOF nanoparticles as pore formers in the fabrication of membranes can create a network of pores	By calibrating the size of the nanoparticles used in membrane construction, it is feasible to enhance membrane porosity and precisely control the mean pore size. This approach can be undertaken without altering the inherent chemical properties of the membrane, such as hydrophilicity and surface charge, maintaining its functional integrity while optimizing its physical structure for improved performance.	[64]
	Creating a selective layer of nanoMOFs on a substrate	Advancements in membrane technology offer the potential to augment the mechanical durability of membranes, precisely tailor surface physicochemical characteristics—including hydrophilicity, electrical charge, and surface roughness—and amplify selectivity, particularly in nanofiltration (NF) and membrane distillation (MD) processes. These enhancements can lead to more resilient and efficient filtration systems.	[65]

**Table 2:** Summary of the latest research results of water treatment MOF composite membranes.

	Membrane Components	Craft	Target Contaminants	References
1	D_6_/TiO_2_/MoS_2_/NiCo-NC/PVDF	Negative pressure assisted method	Pesticides, pharmaceuticals, Personal physical items	[66]
2	MIL-101(Fe)/Cu-POM/IPN	In-situ deposition, pouring	Dye, Drugs	[67]
3	MOF-5/coal-based fiber	Electrospinning	Dye	[68]
4	FS-50/COF(MATPA)-MOFs(Zr)/PDA@PVDF	High pressure induction	Microplastics, dye, pesticides	[69]
5	CoFe-MOF/TiO_2_/PVDF	Non-solvent-induced phase separation	Antibiotic	[70]
6	PAN/Co-MOFs	In-situ growth, electrospinning	Dye	[71]
7	MOF/GO	Vacuum filtration	Dye	[72]
8	MOF/PCL	Solvent/non-solvent methods	Dye	[73]
9	PVA/GO/MOF	Chemical crosslinking and suction filtration	Greasy dirt	[74]
10	Co-CAT-1/PEI/GO	Coating process	Greasy dirt	[75]
11	Bio-MOF-2Me/MMM	Pouring	Cationic dyes	[76]
12	CF/PDA/UiO-66-NH_2_	Grown in situ	Rhodamine B and Pb(II) metalions	[77]
13	Ag(I)-CP/PES	Scraper casting	Dye	[78]
14	MOF/PAN-MIM	In-situ deposition	Bisphenol A	[79]
15	MOF-1a/PVDF	Drip casting method	TNP	[80]

PVDF = Polyvinylidene fluoride; IPN = polymer network; PAN = electrospun polyacrylonitrile; GO = graphene oxide; PCL = porous polycaprolactone; PVA = polyvinyl alcohol; PEI = polyethylenimine; PDA = polydopamine; CF = cotton fabric; PES = sensitive polyether sulfone; TNP = harmful nitro explosive.

### 2.2. Advanced Oxidation

Advanced oxidation processes (AOPs) have emerged as highly effective strategies for contaminant removal, owing to the radicals they generate in situ, such as hydroxyl (·OH), superoxide (·O^2−^), and sulfate (·SO^4−^) radicals. The simplicity of AOP operation, coupled with mild reaction conditions and high efficiency, has garnered significant interest in recent years [81,82]. The potent oxidizing nature of these radicals not only breaks down organic pollutants but also mitigates their toxicity and, in some instances, mineralizes them into CO_2_ [83,84]. Photocatalysis, Fenton and Fenton-like reactions, electrocatalysis, and ozonation, along with various hybrid techniques, are among the AOPs that have seen considerable development for water treatment applications [85,86,87,88]. In the past decade, the application of iron containing MOFs in heterogeneous Fenton reactions has significantly increased due to their high efficiency. Fe MOFs exhibit a higher tendency for electron hole pair recombination, resulting in lower photocatalytic activity. Nowadays, the use of Fe MOFs and semiconductors to prepare composite materials has been designed as a successful strategy to improve charge transfer efficiency. The metal organic framework group of MIL (Lavoisier Materials Research Institute) is one of the most explored categories of MOFs in the field of environmental remediation [89]. Table 3 summarizes some representative studies of MOF (Fe)-based catalysts.

In addition, the latest results show that MOF-derived carbon nanomaterials can avoid the inherent shortcomings of MOF precursors while giving full play to their advantages, and their outstanding performance in the field of AOP water treatment is the focus of research by scientists. For example, C et al. [90] used polydopamine-modified MOF-5-derived carbon as a persulfate activator for aniline air flotation (AFF) degradation, achieving effective oxidation of AAF within 30 min. Wu et al. [91] obtained more active sites by pyrolysis of MOF composites (ZIF-67/NG), which effectively activated peroxymonosulfate tetracycline. Liu et al. [92] using Fe-MOF-derived carbon compounds as catalysts for persulfate oxidative degradation of trichloroethylene, achieved a highest trichloroethylene removal rate of 85.8%. Again, the mechanism involved is the strong oxidation of free radicals, as we have described above, which enables the efficient decomposition of organic pollutants.

**Table 3:** Selective oxidation of MOF-based catalysts.

Catalyst	Organic Pollutant/Removal Efficiency (%)	Co-Existing Substance/Removal Efficiency (%)	Oxidant/ Irrigation	Dominant Mechanism	References
FeCo MOF	CR/95%, RhB/99%, MO/95%, BPA/100%	3-NP/40%, p-BA/50%	PMS	^1^O_2_	[93]
Fe-MOF membrane	BPS/75.7%	BA/-	H_2_O_2_	Size exclusion	[94]
MIL-53(Fe)@ anionicresin	MB/84%	SRB/11%	visible light	Electrostatic interaction	[95]
MIL-53(Fe)@ anionicresin	SRB/73%	MB/59%	visible light	Electrostatic interaction	[95]
Fe-BTC@resin	MB/71%	SRB/12%	visible light	Electrostatic interaction	[96]
Fe-MOF@MIP	SMX/97%	BA/-	PS	MIT	[97]
Fe-MOF-74@MIP	DMP/95%	DEP/80%, DBP/70%, DEHP/70%	PS	MIT	[98]
Fe-MOF- 74@SiO_2_@MIP	DMP/93%	DEP/68%, OG/18%, SMX/23%	PS	MIT	[99]
Zn_4_Co_1_-C	Phenol/100%	BA/1%	PMS	^1^O_2_	[100]
yolk-shell Co/C	BPA/100%	BA/-	PMS	Size exclusion	[101]
Cu/RGO	2,4-DCP/95.8%	BA/0%, CBZ/10%, IBU/30%	PDS	Cu(III)	[102]
Fe/Fe_3_O_4_@rGO	BPA/90%, BPF/60%, MeP/12%, p-BA/13%, 2,4-DCP/100%, CP/30%, BPS/12%, phenol/12%	-	PDS	Hydrophobicity	[103]

Note: BTC = 1,3,5-tricarboxylic acid; MIT = molecularly imprinted technology; RhB = rhodamine B; BPA = bisphenol A; BPS = bisphenol S; MB =methylene blue; SMX = sulfamethoxazole; DMP = dimethyl phthalate; 2,4-DCP = 2,4-dichlorophenol; BPF = bisphenol F; p-BA = 4-hydroxybenzaldehyde; CP = 4-chlorophenol; 3-NP = 3-nitrophenol; BA = benzoic acid; IBU = ibuprofen.

### 2.3. Other MOF-Based Water Treatment Strategies

#### 2.3.1. Acts as a Flocculant to Remove Harmful Algae

In the quest for advanced water treatment solutions, the MIL series of metal–organic frameworks (MOFs) has emerged as particularly promising. Notably, MIL-101 distinguishes itself through its exceptional chemical robustness in various solvents, ease of production, biological compatibility, extensive surface area, and cost-effectiveness [104,105,106]. For instance, a groundbreaking application proposed by Li et al. [107] leverages MOFs, specifically NH_2_-MIL-101(Cr), as flocculants to address the pervasive issue of harmful algal blooms. Their research revealed that this nanoscale, amine-functionalized chromium-based MOF is particularly effective against Microcystis aeruginosa—a dominant and ecologically damaging algae species. This MOF exhibits superior algal removal capabilities, surpassing those of traditional flocculants across a spectrum of environmental conditions, demonstrating a powerful potential for water purification processes. The mechanism behind NH_2_-MIL-101(Cr)’s effectiveness is believed to be twofold: firstly, the MOF particles aggregate and attach to the algae cells; subsequently, they co-precipitate, removing the algae from the water. This not only clears the immediate contamination but also helps restore water quality and maintain ecological balance. Despite these advantages, it is imperative to consider the broader implications of introducing such materials into the environment. Potential impacts on water quality, the ecosystem, and public health need thorough assessment to mitigate any risks associated with their use. Responsible application of MOFs, with careful monitoring and adherence to safety guidelines, is essential to harness their benefits without compromising environmental integrity and human well-being.

#### 2.3.2. Inhibit the Growth of Algae

**Figure 3:**
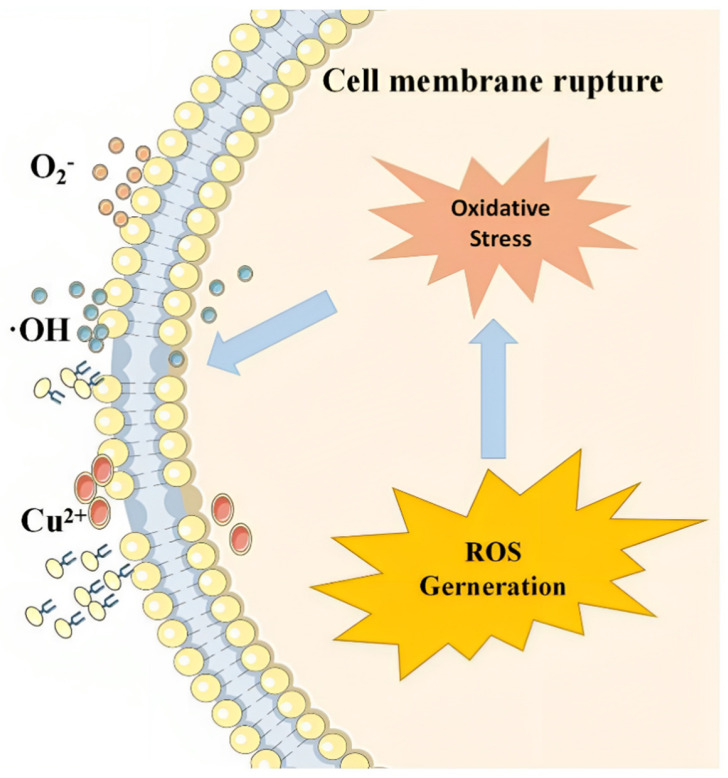
The principle of inhibiting the growth of *M. aeruginosa* by Fe_3_–O_4_BC@Cu–MOF–74 [89].

Exploiting the multifaceted advantages of MOFs, various studies have probed their potential in controlling algal proliferation [108,109,110]. For example, Fan et al. [111] showcased the remarkable algae-inhibiting capabilities of Cu-MOF-74, recording a 75.5% removal rate of algae within 120 h. This significant reduction is partially attributed to a 56% decrease in chlorophyll a content, indicating impairment of the photosystem due to diminished chlorophyll a and phycobiliprotein levels. This damage stems from the sustained release of copper ions (Cu^2+^) from Cu-MOF-74, which serve as a metal ion reservoir, inducing reactive oxygen species (ROS) accumulation within the algal cells. This oxidative stress leads to lipid peroxidation, evidenced by the loss of oxidation balance, decreased superoxide dismutase (SOD) activity, and increased catalase (CAT) activity in the algal cells (Figure 3). In addition, the generation of hydroxyl radicals by Cu-MOF-74 further exacerbates cellular damage, culminating in the death of Microcystis aeruginosa. Consequently, the concentration of extracellular organic matter in the water is observed to rise. These findings illuminate the efficacy of copper-based MOFs, not just as inhibitors but also as agents of removal for harmful algal species.

### 2.4. Sensing Monitoring and Detection

The myriad water treatment methodologies utilizing MOFs, as outlined above, highlight the versatility of these materials. Concurrently, the evolution of sensing technologies has paved the way for the use of MOFs in the detection of water quality, adding an additional layer of utility to their application in water management.

Despite the promising nature of these cutting-edge MOF-based technologies, it is important to note that their implementation, whether standalone or in synergy with established methods, is still undergoing preliminary assessment. Nevertheless, available research data suggest that these novel approaches—whether applied individually or in tandem—have already demonstrated effective pollutant mitigation capabilities [112]. In the following sections, we will delve into three pivotal studies that underscore the use of MOFs in both the remediation and monitoring aspects of water treatment, thereby painting a comprehensive picture of their role in ensuring water safety and purity.

#### 2.4.1. Fluorescence Detection

**Figure 4:**
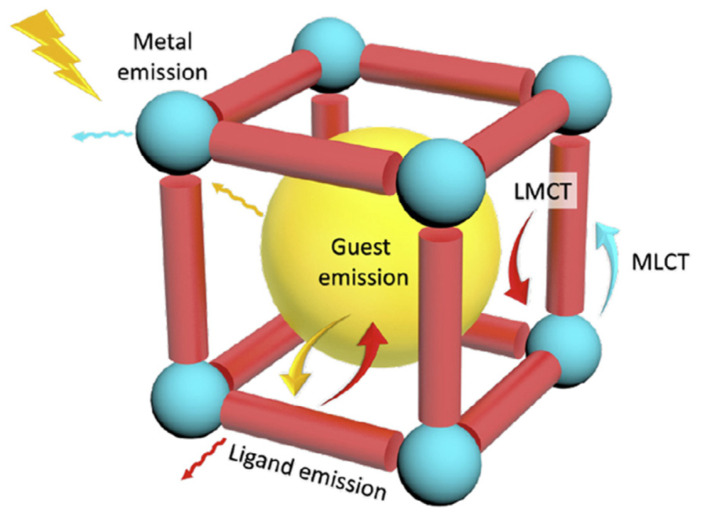
Schematic diagram showing the different sources of fluorescence signals in MOF-based fluorescence sensors [133].

A significant body of early research on MOF-based fluorescence sensors has focused on detecting key pollutants known for their adverse impact on water quality, such as metal ions, anions, and nitroaromatic compounds. These compounds are adept at quenching fluorescence through processes like internal filtering effects (IFE) and photoinduced electron transfer (PET) [113,114]. MOF-based sensors distinguish themselves from other fluorescence sensors crafted from small molecules or conjugated polymers by offering superior chemical tunability, which facilitates precise and efficient host–guest recognition [115,116,117,118,119,120,121,122,123]. Moreover, the inherent structure of MOFs, laden with numerous π and n electrons, is conducive to producing robust and diverse fluorescence signals [124,125,126]. This, combined with their capacity to be easily modified and decorated onto various substrates, positions MOFs as particularly promising candidates for fluorescence-based sensing applications [127,128,129,130,131]. Illustrating their potential, Huang et al. [132] reported on the solvothermal synthesis of an Al^3+^ MOF, denoted as CAU-1-(OH)_2_, featuring ligands that strongly coordinate with Bi^3+^ due to the hydroxyl and carboxyl groups. This affinity of bismuth for oxygen, which is greater than that of the MOF’s metal center, leads to the replacement of Al with Bi, resulting in fluorescence quenching. CAU-1-(OH)_2_ showcases the ability to detect Bi^3+^ in water with a remarkably fast response time of 24 s and a detection limit of 2.16 μM. Although its sensitivity to pH may influence detection, this sensor serves as a valuable prototype for future sensor development aimed at Bi^3+^ and possibly other metal ions [133]. Figure 4 shows the different fluorescence signal sources in MOF-based fluorescence sensors.

#### 2.4.2. Electrochemical Sensing

In addition to the aforementioned fluorescence detection, the advancements in nanotechnology and functional materials have catalyzed the evolution of electrochemical detection techniques [134,135]. Leveraging their unique structures and properties, these materials provide a solid framework for developing sophisticated electrochemical sensors, particularly for the surveillance of heavy metal ions in various environments [136,137,138,139]. Table 4 offers a comprehensive overview of MOF-based electrochemical sensors designed for the detection of transition metal ions. It reveals that the predominant sensing mechanism in these applications is the cation exchange between the metal ions and the MOFs. This exchange mechanism plays a crucial role in the sensor’s ability to identify and quantify specific metal ions, underscoring the versatility and effectiveness of MOFs as a key component in environmental monitoring strategies.

The efficacy of sensors is closely tied to their sensitivity toward the targeted analyte, which in turn is intricately linked to the electrochemical kinetics within the analyte. A predominant challenge with metal–organic frameworks (MOFs) is their tendency to decompose in aqueous media [167], a particular concern given that electrochemical sensing operations often occur in such environments. The hydrolytic stability of MOFs is thus a paramount factor for their effective use in electroanalysis, as water molecules can disrupt the metal–ligand bonds, leading to the formation of metal hydroxides or oxides [168]. Moreover, it is well-established that many MOFs exhibit limited electrical conductivity, which is less than ideal for sensor applications [169]. To address this, various strategies have been explored to enhance both the electrical conductivity and stability of MOFs during electrochemical processes. With these improvements, we anticipate a corresponding increase in the sensitivity of electrochemical sensors utilizing MOF-based materials. Observations indicate that MOFs with nitrogen-containing ligands show enhanced hydrolytic stability compared to those with carboxylate-based ligands. This suggests a pathway to mitigate hydrolytic instability issues—by opting for MOFs with nitrogenous ligands. Additionally, selecting metal cations with higher oxidation states for the nodes and employing stable organic linkers can lead to the development of chemically robust MOFs, capable of maintaining their integrity in challenging conditions [170].

#### 2.4.3. Biosensing

**Figure 5:**
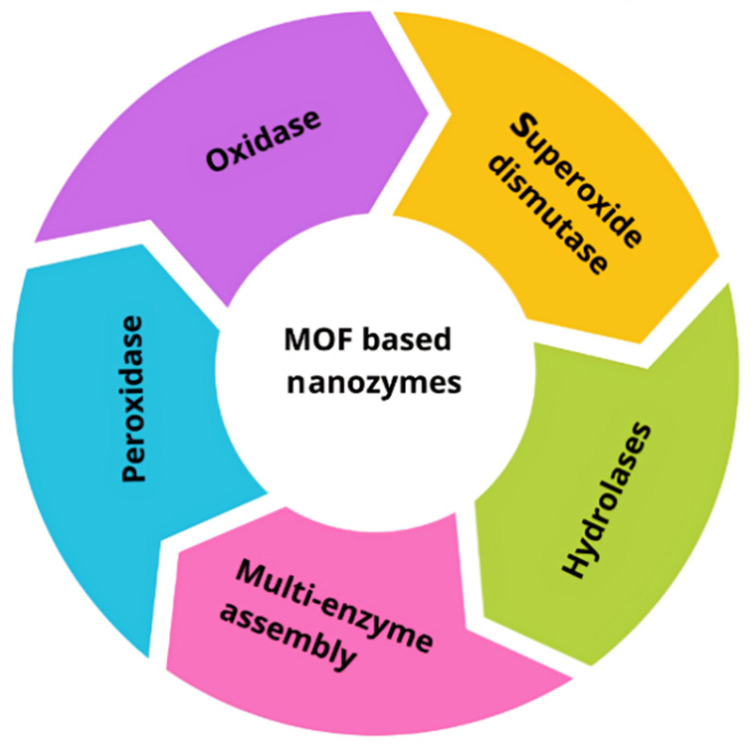
Typical MOFs are explored as different activities of nanozymes [152].

MOF-based biosensors are at the forefront of contemporary analytical science, with extensive research dedicated to the detection of a broad range of analytes [171]. The inherent structural functionality of MOFs—owing to the presence of groups such as -NH_2_ or -COOH within their linkers—facilitates critical interactions like p-p stacking, hydrogen bonding, and electrostatic forces with probe biomolecules. This makes MOFs an exceptional substrate for biosensing applications. The emerging class of bimetallic MOFs is garnering attention in electrochemical biosensing due to their enhanced catalytic activities. For instance, the bimetallic ZrHf-MOFs/carbon dots composite developed by Gu et al. [172] demonstrated exceptional sensing performance for HER-2 cells, underscoring the potential of combining diverse metal elements to introduce new functionalities and achieve synergistic effects that boost the biocatalytic properties of MOFs. Figure 5 shows the different activities of typical MOFs explored as nanomasses [152].

However, despite groundbreaking developments and impressive research outputs, the field of MOF-based biosensors is still nascent, and several challenges beckon resolution: (1) Synthesizing MOF-based biosensors that meet required efficiency benchmarks remains an ongoing challenge [173,174,175,176]. (2) As the majority of MOFs are synthesized using organic solvents, their stability in aqueous environments warrants thorough investigation. (3) The creation of unique MOF-based biosensors, characterized by distinctive magnetic, thermal, and electrochemical properties, is still an objective to be realized [177,178,179,180]. (4) While MOFs can function as independent catalysts, their activity levels are generally lower than natural enzymes, likely due to the intrinsic reactivity limitations of pristine MOFs. This disparity signifies the urgent need to design new MOF-based nanozymes with significantly higher activity profiles.

## 3. Large-Scale Preparation of MOF

Despite notable advancements in the synthesis and application of MOFs, a key concern revolves around their potential for industrialization and practical applications, particularly in high-volume use cases such as water treatment in environmental applications. Over time, the concerted efforts of material scientists and chemical engineers have optimized the synthesis of functional MOFs, making it more facile and cost-effective [46]. A range of large-scale preparation methods has been developed and considered, encompassing traditional approaches like electrochemical [24], microwave [25] and mechanochemistry [26] approaches and more recent routes like the spray dryer [27], flow chemistry [28] and high-pressure homogenization (HPH) [29]. Within this array of methods, two newly investigated approaches are delineated in the following section.

### 3.1. Spray-Drying (SD)

**Figure 6:**
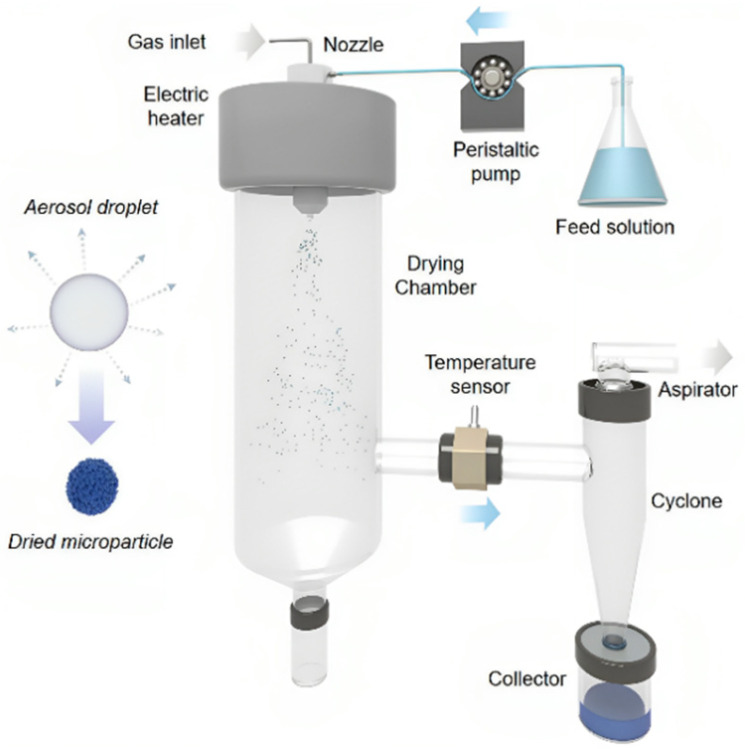
Schematic of the general spray drying setup [27].

The spray drying (SD) process has been a cornerstone in industrial manufacturing for a myriad of sectors for many years [181,182,183,184]. This technique involves the atomization of a liquid or slurry into a hot gas to rapidly produce dispersed powder particles, as depicted in Figure 6. SD stands out for its ability to facilitate rapid, continuous, and scalable production of dry microspherical powders through a single-step process. The result is a reduction in fabrication costs and production times when compared to more conventional powder production methods. Since 2013, pioneering work by Arnau Carne-Sánchez and colleagues has broadened the array of chemical processes achievable within aerosol droplets, extending beyond simple precipitation to sophisticated coordination and covalent chemistries [27]. Their research has showcased spray drying as a viable and efficient technique for the synthesis of crystalline, porous, nanostructured materials. This includes not only MOFs but also Covalent Organic Frameworks (COFs) and a variety of their composites, offering a versatile platform for material development [185]. Table 5 summarizes the common MOFs produced by spray drying with different feeding methods. The reason for choosing different methods is because before the atomization step, the precursor feed is introduced into the nozzle via simple peristaltic-pump tubing. This direct injection strategy is straightforward and convenient for precursor solutions that do not undergo any undesired reactions before spray drying. However, some precursor solutions are unstable because they contain highly reactive reagents. In these cases, the precursor solutions must first be mixed shortly before or immediately after atomization, which can be achieved through different introduction methods.

In the synthesis of MOFs via spray drying, critical parameters that require meticulous management include (1) the feed rate, which determines the amount of liquid precursor introduced into the drying system; (2) the atomization flow rate, affecting the size of liquid droplets formed; (3) the inlet temperature (Tinlet), which is pivotal for drying the aerosol droplets efficiently; and (4) for continuous flow-assisted synthesis, the coil-flow reactor temperature (Tcoil) needs careful adjustment. Researchers are tasked with striking an optimal balance among these factors. For example, an increased feed rate can boost production; however, it necessitates greater energy input for atomization and drying, thus raising operational costs [27,185,192,193,194].

It is evident that obtaining optimal conditions for producing specific MOFs requires experimental methods based on real and reliable data and phenomena. However, due to limitations in experimental technology, equipment, measurement methods, and other factors, experimental costs are high, cycles are long, and data fluctuations are significant. The spray drying tower, during the experiment, is relatively closed and is considered a “black box”. The phenomena in the tower are challenging to observe, and obtaining specific information about its flow field is difficult [195,196]. In recent years, the computer hardware configuration has been constantly upgraded, and various hydrodynamic calculation models have been constantly improved. Numerical simulation provides a new way for in-depth research on hydrodynamics in spray drying towers, which is expected to solve the above problems. Therefore, relevant research on numerical simulation of MOF spray drying production needs to be promoted as soon as possible, which plays an important role in optimizing spray drying process to prepare MOFs in the long run.

### 3.2. High-Pressure Homogenization (HPH)

High-pressure homogenization (HPH) technology is an established method widely utilized across various industries, including biological, pharmaceutical [197], food [198,199,200,201], chemical [202], and industrial polymer synthesis [203]. It is commercially viable, cost-efficient, and straightforward to operate at room temperature. It is characterized by low energy requirements, high production efficiency, and the capacity for continuous operation [204]. In the realm of HPH, reactants are efficiently dispersed within a solvent, significantly outperforming conventional methods such as mechanochemical synthesis or twin-screw extrusion in terms of mass and thermal transfer. The technology also supports the continuous synthesis process through the sequential injection of reactants, which is a stark contrast to traditional batch methods like hydrothermal/solvothermal, sonochemical, and microwave-assisted synthesis [29,205].

**Figure 7:**
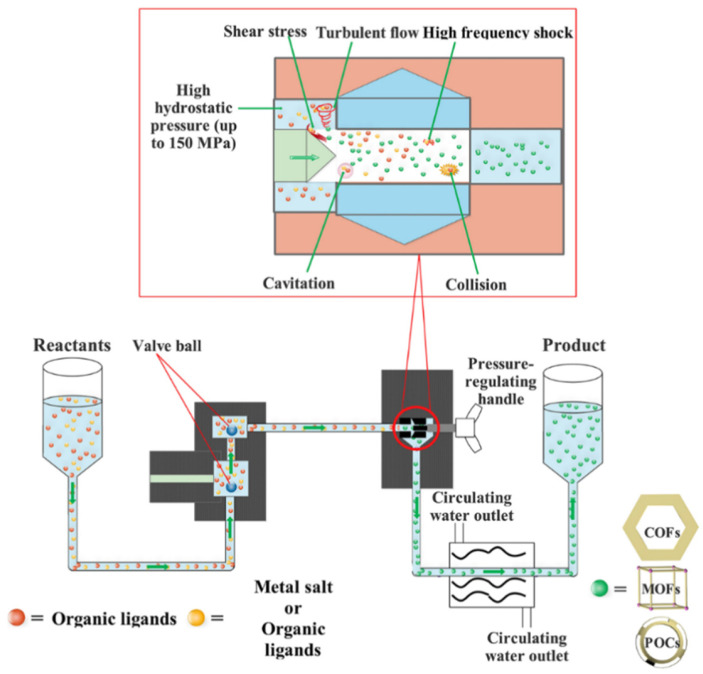
High pressure homogenization strategy for synthesizing crystalline porous materials [29].

Liu et al. [29] have further innovated within this space, pioneering a novel HPH-based technique for the large-scale, continuous synthesis of crystalline porous materials. The effectiveness of HPH approach in synthesizing these crystalline porous materials can be attributed to the following reasons: (1) the homogenization process induces cavitation within the pipes, potentially creating a local vacuum that protects organic ligands from partial oxidation and thereby enhances the reaction [204]; (2) the solvent environment under HPH conditions is optimized for reactant mass transfer, thereby improving the yield and consistency of the resulting products [206,207]; (3) the intense mechanical forces—shear stress, collision, high-frequency shock, and turbulent flow—induced by HPH significantly accelerate the formation of structures such as Covalent Organic Frameworks (COFs), MOFs, and Porous Organic Cages (POCs); (4) this method promises bulk production of these materials through consecutive reactant injections [208] (Figure 7). Therefore, HPH technology addresses the limitations of conventional and other reported synthesis methods by increasing yield and efficiency while reducing energy consumption and simplifying the production process. It also alleviates issues seen with mechanochemical and twin-screw extruder approaches, such as low crystallinity, inadequate mass/thermal transfer, limited reproducibility, and batch production constraints.

Despite the advancements in mass production techniques, the synthesis of new metal–organic frameworks (MOFs) remains largely limited to certain types, hindering the diversity and innovation within this field. This bottleneck in production technology underscores a crucial need for the development of more versatile and robust mass production methods. Such advancements would not only broaden the spectrum of MOF types that can be synthesized on a commercial scale but also streamline the integration of these materials into various applications. There is a clear anticipation within the industry and academia for innovative manufacturing processes that can match the complexity of MOF chemistry with the practicalities of commercialization, thereby unlocking the full potential of MOFs in commercial applications.

## 4. Summary and Prospect

In summary, this discussion outlines prevailing MOF-based approaches applied in wastewater treatment to date, alongside two developed methods for large-scale MOF preparation. The significant impact of MOFs in this domain is rooted in four key attributes: (1) Enhanced stability: select MOFs maintain their integrity under a wide pH range, underscoring their viability in diverse environmental contexts. (2) Versatile ligand modifications: the adaptability in customizing MOFs through various functionalization techniques enables the integration of functional groups, enhancing the MOFs’ performance. (3) Functional metal sites: the incorporation of coordinatively unsaturated metal sites within MOFs performs a dual role, anchoring pollutants for removal and serving as catalytic centers for their breakdown. (4) Multifaceted functionalization: employing a range of functionalization strategies harnesses their collective strengths, bolstering the efficiency of the pollutant degradation process.

Despite these promising features, widespread adoption of MOFs is hampered by challenges related to the materials’ inherent properties and the need for better process flows. To pave the way for the wider practical application of MOFs, we present several strategic considerations: (1) Preliminary design and theoretical prediction: engage in rigorous initial design and leverage theoretical models to guide efficient large-scale synthesis. (2) Selection of organic linkers: opt for biocompatible, bio-inspired, and biodegradable organic linkers to enhance the environmental compatibility of metal–organic frameworks (MOFs). (3) Choice of reaction medium: prioritize water or green solvent systems to align with sustainable manufacturing practices. (4) Minimization of bulk organic solvents: minimize the use of organic solvents to sup-port eco-friendly MOF production methods. Additionally, the synthesis, reuse, and regeneration of MOFs must be considered holistically, incorporating techniques such as thermal and vacuum treatments, solvent exchange, supercritical CO_2_ activation, freeze drying, and chemical activation. It is essential to establish robust protocols for recycling, regeneration, and the management of MOF waste, extending their utility across a broader spectrum of applications. A critical examination of solvent roles and choices in synthesis is paramount to forging effective, sustainable strategies for MOF production and application.

## Figures and Tables

**Table 4:** Recent overview of MOF-based sensors for transition metal ions and their mechanism of sensing.

Transition Metal Ion	MOF-Based Sensor	Detection Mechanism	References
Cr^+3^	3D Ca-MOF	Competitive absorption mechanism	[140]
Cr^+3^	Eu-MOF	Cation exchange quenching mechanism	[141]
Cr^+3^	Eu-MOF	Cation exchange quenching mechanism	[142]
Cr^+3^	Tb-MOF	Cation exchange quenching mechanism	[142]
Cr^+6^	MOF-199	Oxidoreductase	[143]
Cr^+3^	MIL53-L	LLCT mechanism	[144]
Cr^+3^	BPEI-CQDs/ZIF-8-MOFs	Cation exchange quenching effect	[145]
Cr^+3^	[NH_4_]_2_ [ZnL]·6H_2_O	Host-guest Interaction	[146]
Cr^+3^	(Lin et al.)^n^	Cation exchange quenching mechanism	[147]
Cr^+3^	Cd-MOF-74	PET/FRE mechanism	[148]
Cr^+3^	Zn-based HPU-1	Zn-based HPU-1	[149]
Cr^+3^	2D[Zn_2_(TPC_4_A)(DMF)(H_2_O_4_)]_3_H_2_O	Cation exchange quenching mechanism	[150]
Mn^+2^	MOFs [Zn (dbp)]^n^	Competitive absorption	[151]
Mn^+2^	[Cd (dbp) (H_2_O)]2H_2_O·CH_3_CN]^n^	Competitive absorption	[152]
Mn^+2^	[NH_4_]_2_ [ZnL]·6H_2_O	Host-guest Interaction	[146]
Mn^+2^	MOF-525	Cation exchange quenching mechanism	[153]
Mn^+2^	PCN-222-Pd	Cation exchange quenching effect	[144]
Fe^+3^	3D [[(CH_3_)2NH_2_]_2_ [Zn-(TNC_4_A)][[(CH_3_)2NH_2_]_2_[Zn-(TNC_4_A)][[(CH_3_)2NH_2_]2 [Zn-(TNC_4_A)]·4H_2_O	Cation exchange quenching mechanism	[150]
Fe^+3^	NNU-1	Cation exchange quenching effect	[154]
Fe^+3^	[Zn_2_(OBA)_2_(BPTP)]	Competitive absorption quenching effect	[155]
Fe^+3^	[Ni(OBA)_2_(BPTP)_2_(H_2_O)_2_]	Competitive absorption quenching effect	[155]
Fe^+3^	[Cd_2_(OBA)_2_(BPTP) (H_2_O)]	Competitive absorption quenching effect	[155]
Fe^+3^	[Cd(L) (BPDC)](H_2_O)_2_	Competitive absorption quenching effect	[155]
Fe^+3^	[Cd(L)(SDBA)(H_2_O)](H_2_O)_0_._5_	Competitive absorption quenching effect	[155]
Fe^+3^	Cd-MOF	FRET/PET mechanism	[156]
Fe^+3^	BUT-14	RET/FRET mechanism	[156]
Fe^+3^	BUT-15	RET/FRET mechanism	[156]
Fe^+3^	UMCM-1-NH_2_	Fluorescence quenching mechanism	[156]
Fe^+3^	[Cd (5-asbaz (bimb)]^n^	FRET/PET mechanism	[156]
Fe^+3^	3D Tb-MOF	FRET/PET mechanism	[157]
Fe^+3^	Zirconium MOF	FRET/PET mechanism	[158]
Fe^+3^	Zn-MOF	LLCT mechanism	[159]
Zn^+2^	([Ln (PDA)_3_Mn_1.5_(H_2_O)3]·3.25H_2_O	PET/FRET mechanism	[160]
Pd^+2^	NH_2_-MIL-53(Cr)	PET/FRET mechanism	[160]
Cd^+2^	Zn-MOF	LMCT mechanism	[161]
Hg^+2^	ZnMOF	Cation exchange quenching effect	[162]
Hg^+2^	EuMOF	PET/FRET mechanism	[163]
Hg^+2^	RuMOF	PET/FRET mechanism	[164]
Hg^+2^	Eu/IPA CPNPs	PET/FRET mechanism	[162]
Lanthanides	{[GdIII_2_L_64_)Mn(H_2_O)_6_]·XH_2_O}n	Cation exchange quenching effect	[165]
Lanthanides	CdMOFs	PET/FRET mechanism	[165]
Lanthanides	MgMOF	PET/FRET mechanism	[165]
Miscellaneous Transition Metal Ions	Eu^3+^@MIL-121	PET/FRET mechanism	[166]
Miscellaneous Transition Metal Ions	UiO-bpydc	PET/FRET mechanism	[166]
Miscellaneous Transition Metal Ions	Eu-bpydc	PET/FRET mechanism	[166]

Note: H_2_dbp = 4′-(4-(3,5-dicarboxylphenoxy)phenyl)-4,2′:6′,4′′-terpyridine; BPEI-CQD = poly-(ethylenimine)-capped carbon; DMF = Dimethyl formamide; TPC = terephthalyl chloride; dbp = dibutyl phthalate; bimb = 4,4′-Bis(1-imidazolyl)biphenyl; BPTP = 3,5-bis(5-(pyridin-4-yl)thiophen-2-yl)pyridine)[Ni(OBA)_2_(BPT P)_2_(H_2_O)_2_] and [Cd_2_(OBA)_2_(BPTP) (H_2_O); PDA = p-Phenylenediamine; BPDC = 4,4′-biphenyldicarboxylic acid; SDBA = 4,4′-sulfonyldibenzoic acid; bpydc = 2,2′-bipyridine 5,5′-dicarboxylic acid.

**Table 5:** Summary of MOF and coordination polymer synthesized by spray drying.

Catalysts	Precursors	Solvent (s)	T_inlet_ (T_coil_) [°C]	Yield [%]	S_BET_ [m^2^/g]	References
HKUST-1	Cu(NO_3_)_2_, BTC	DMF/EtOH/H_2_O	180	70	1260	[27]
Cu-BDC	Cu(NO_3_)_2_, BDC	DMF	180	70	543	[27]
NOTT-100	Cu(NO_3_)_2_, BPTC	DMF/H_2_O	180	54	1140	[27]
MOF-14	Cu(NO_3_)_2_, BTB	DMF/EtOH/H_2_O	180	30	-	[27]
Zn-MOF-74	Zn(NO_3_)_2_, DHBDC	DMF/H_2_O	180	50	-	[27]
Mg-MOF-74	Mg(NO_3_)_2_, DHBDC	DMF/EtOH/H_2_O	180	35	-	[27]
Ni-MOF-74	Ni(NO_3_)_2_, DHBDC	DMF/EtOH/H_2_O	180	40	-	[27]
MIL-88B	FeCl_3_,NH_2_-BDC	DMF/MeOH/H_2_O	180	27	-	[27]
ZIF-8^a^	Zn(OAc)_2_, 2-MIM	H_2_O	180	-	1634	[186]
ZIF-67^a^	Co(OAc)_2_, 2-MIM	H_2_O	180	-	1861	[186]
Zn/Co-ZIF^a^	Zn(OAc)_2_, Co(OAc)2,2-MIM	H_2_O	180	-	1746	[186]
[Fe(NH_2_trz)_3_]Br_2_·nH_2_O	FeBr_2_, NH_2_-TRZ		90	-	-	[187]
[Fe(NH_2_trz)_3_](BF_4_)_n_	FeBr_2_, NH_2_-TRZ	EtOH; H_2_O	90	-	-	[187]
[Fe(Htrz)_2_(trz)](BF_4_)_n_	Fe(BF_4_)_2_, HTRZ	EtOH; H_2_O	90	-	-	[187]
Tb0.914Eu0.086-PDA	Tb(NO_3_)_3_, Eu(NO_3_)_3_, PDA	DMF/H_2_O	180	55		[188]
MIL-88A	FeCl_3_, FUM	DMF/MeOH/H_2_O	180	40	-	[27]
MOF-5	Zn(OAc)_2_, BDC	DMF	180	60	1215	[27]
IRMOF-3	Zn(OAc)_2_, NH_2_-BDC	DMF	180		70	[27]
ZIF-8	Zn(OAc)_2_, 2-MIM	H_2_O	180	10	941	[27]
Cu-PB	Cu(NO_3_)_2_, K_3_Co(CN)_6_	H_2_O	180	20	617	[27]
SIFSIX-3-Co	CoSiF_6_, PYZ	MeOH	85	44	-	[189]
SIFSIX-3-Ni	NiSiF_6_, PYZ	MeOH	85	-	-	[189]
SIFSIX-3-Cu	CuSiF_6_,PYZ	MeOH	85	55	-	[189]
SIFSIX-3-Zn	ZnSiF_6_, PYZ	MeOH	85	57	-	[189]
SIFSIX-1-Zn	ZnSiF_6_, BPY	MeOH	85	40	-	[189]
TIFSIX-1-Cu	Cu(NO_3_)_2_, BPY	MeOH	130	79	-	[189]
UiO-66	ZrCl_4_, BDC	DMF/H_2_O	180 (115)	70	1106	[190]
UiO-66-NH_2_	ZrCl_4_, NH_2_-BDC	DMF/H_2_O	180 (115)	67	752	[190]
UiO-66-NO_2_	ZrCl_4_, NO_2_-BDC	acetic acid/H_2_O	180 (115)	62	679	[190]
UiO-66-Br	ZrCl_4_, Br-BDC	DMF/H_2_O	180 (115)	68	527	[190]
UiO-66-(OH)_2_	ZrCl_4_, (OH)_2_-BDC	DMF/H_2_O	180 (115)	81	401	[190]
UiO-66-acetamido	ZrCl_4_, acetamido-BDC	DMF/H_2_O	180 (115)	51	586	[190]
UiO-66-1,4-NDC	ZrCl_4_, 1,4-NDC	DMF/H_2_O	180 (115)	45	431	[190]
UiO-66-2,6-NDC	ZrCl_4_, 2,6-NDC	DMF/H_2_O	180 (115)	49	557	[190]
Fe-BTC/MIL-100	Fe(NO_3_)_3_, BTC	DMF	180 (135)	78	1039	[190]
Ni_8_(OH)_4_(H_2_O)_2_(L)_6_	Ni(OAc)_2_, PCA	DMF/H_2_O	180 (100)	60	377	[190]
UiO-66-NH_2_	ZrOCl_2_, NH_2_-BDC	acetic acid/H_2_O	150 (90)	64	1261	[191]
Zr-fumarate	ZrOCl_2_, FUM	acetic acid/H_2_O	140 (90)	58	664	[191]

Crystallized in a solvent after spray drying. BTC, trimesic acid; BDC, terephthalic acid; BPTC, biphenyl-3,3′,5,5′-tetracarboxylic acid; BTB, 1,3,5-tris(4-carboxyphenylbenzene); DHBDC, 2,5-dihydroxy-terephthalic acid; 2-MIM, 2-methylimidazole; R-TRZ, 4-R-substituted-1,2,4-triazole; PDA, 1,4-phenylenediacetic acid; FUM, fumaric acid; PYZ, pyrazine; BPY, 4,4′-bipyridine; NDC, naphthalenedicarboxylic acid; PCA, 1H-pyrazole-4-carboxylic acid.

## Data Availability

Not applicable.
